# Multi-drug resistant spinal tuberculosis-epidemiological characteristics of in-patients: a multicentre retrospective study

**DOI:** 10.1017/S0950268820000011

**Published:** 2020-01-27

**Authors:** S. Yang, Y. Yu, Y. Ji, D. J. Luo, Z. Y. Zhang, G. P. Huang, F. Y. He, W. J. Wu, X. P. Mou

**Affiliations:** 1 Department of Orthopedics, Southwest Hospital, Third Military Medical University, Chongqing, China; 2 Department of Orthopedics, Hospital of Chengdu University of Traditional Chinese Medicine, Chengdu, Sichuan Province, China; 3 First Department of Orthopedics, the People's Hospital of Jian yang, Jian yang, China

**Keywords:** Anti-tubercular chemotherapy, drug resistance, epidemiology, spinal tuberculosis, surgery

## Abstract

Tuberculosis (TB) is the leading cause of death among infectious diseases. China has a high burden of TB and accounted for almost 13% of the world's cases of multi-drug resistant (MDR) TB. Spinal TB is one reason for the resurgence of TB in China. Few large case studies of MDR spinal TB in China have been conducted. The aim of this research was to observe the epidemiological characteristics of inpatients with MDR spinal TB in six provinces and cities of China from 1999–2015. This is a multicentre retrospective observational study. Patients' information was collected from the control disease centre and infectious disease database of hospitals in six provinces and cities in China. A total of 3137 patients with spinal TB and 272 patients with MDR spinal TB were analysed. The result showed that MDR spinal TB remains a public health concern and commonly affects patients 15–30 years of age (34.19%). The most common lesions involved the thoracolumbar spine (35.66%). Local pain was the most common symptom (98.53%). Logistic analysis showed that for spinal TB patients, reside in rural district (OR 1.79), advanced in years (OR 1.92) and high education degree (OR 2.22) were independent risk factors for the development of MDR spinal TB. Women were associated with a lower risk of MDR spinal TB (OR 0.48). The most common first-line and second-line resistant drug was isoniazid (68.75%) and levofloxacin (29.04%), respectively. The use of molecular diagnosis resulted in noteworthy clinical advances, including earlier initiation of MDR spinal TB treatment, improved infection control and better clinical outcome. Chemotherapy and surgery can yield satisfactory outcomes with timely diagnosis and long-term treatment. These results enable a better understanding of the MDR spinal TB in China among the general public.

## Introduction

Tuberculosis (TB) has been a serious infectious disease in developing countries, and spinal TB is the most common bone and joint TB, accounting for about 50% of cases [1]. Spinal TB often leads to irreversible neurological injury, including paralysis, resulting in serious social and economic problems [2]. In recent years, with the increase in the number of HIV-infected patients, TB infections have gradually increased across the world. According to a 2018 research report by the World Health Organization (WHO) [3], 10 million people worldwide developed TB disease in 2017 and there was an estimated 1.6 million TB patients deaths at the same year. China is the largest developing country, which had the second highest burden of TB in the world. During the past few decades, great effort has been made to control the spread of TB in China. However, drug resistant tuberculosis (DR-TB) is becoming more serious [4].The latest global TB report 2018 pointed out that the number of new TB patients in China reached 889 000, of which DR-TB accounted for 7.1% [3]. Multi-drug resistant tuberculosis (MDR-TB) is resistant to most first-line anti-TB drugs, the current standard chemotherapy regimen is not effective, and the disease gradually develops into refractory, recurrent TB, which seriously affects the prognosis. Although current diagnostic imaging devices provide additional information, diagnosis and treatment still face challenges due to the slow progress in clinical features, which lead to neurological complications and spinal deformities. Early diagnosis and treatment are the key to avoiding this chronic disability.

With the development of molecular biology, more and more molecular diagnostic techniques have been applied to clinics [5]. Diagnostic ability has made great progress, but there are still some defects in the sensitivity and specificity. The significance of the diagnosis of DR-TB is further discussed.

The best treatment regimen for MDR spinal TB remains controversial, especially with regard to the choice of chemotherapy and duration (chemotherapy alone or combined with surgery). Anti-TB drug chemotherapy is the key and foundation of curing spinal TB, with surgery being an adjuvant means of treatment [6]. However, improper dosing, inadequate duration of treatment and inappropriate selection of candidates for chemotherapy have not only resulted in the resurgence of TB but also led to the most dreadful consequence of multidrug resistant strains [7].

In spinal TB, especially MDR spinal TB, the diagnosis, chemotherapy, surgery and prognosis are still controversial. Meanwhile, a large sample and multicentre study of the clinical features, diagnosis and treatment of MDR spinal TB is relatively rare. Therefore, we retrospectively analysed the epidemiological characteristics, clinical characteristics and efficacy of MDR spinal TB in four provinces with TB high-risk (Shaanxi, Guangxi, Sichuan and Gansu) and two municipalities (Chongqing and Beijing) in the last 17 years.

## Materials and methods

### Study sites

Chongqing municipality is a city of 30 484 300 located in southwest China, with a rural population of 12 100 200. According to the National TB Survey in 2015, the TB prevalence (including urban and rural areas) was 75.0 per 100 000. In this study, we selected the largest comprehensive hospital in Chongqing (Southwest Hospital affiliated to Third Military Medical University) and control disease centre (CDC). Beijing municipality, the capital of China, is a city of 21 729 000 located in north China, with a rural population of 2 952 000. The TB prevalence was 32.0 per 100 000. We selected the spinal TB treatment centre of PLA (309 Hospital of PLA) and CDC. Shaanxi is a province of 38 126 200 located in northwest China, with a rural population of 17 027 200. The TB prevalence was 52.6 per 100 000. We selected the largest orthopaedic hospital (Hong Hui Hospital Affiliated to Xi'an Jiao Tong University) and CDC. The Guangxi autonomous region is a province of 55 791 200 located in south China, with a rural population of 33 221 200. The TB prevalence was 96.4 per 100 000. We selected two general hospitals (Sixth Affiliated Hospital of Guangxi Medical University and Nanning second people's Hospital) and CDC. Si Chuan is a province of 82 620 000 located in southwest China, with a rural population of 41 963 000. The TB prevalence was 62.8 per 100 000. We selected two hospitals in a high-prevalence area (The Affiliated hospital of southwest medical university and Liangshan Yi autonomous prefecture people's hospital) and CDC. Gan Su is a province of 25 907 800 located in west of China, with a rural population of 15 109 400. The TB prevalence rate was 52.2 per 100 000. We selected a polyclinic hospital (The Lanzhou general hospital, Lanzhou Military Command of CPLA) and CDC.

### Study design and patients records

This research was approved by the Ethics Committee of the People's Hospital of Jianyang. This epidemiological study used patients' information from the CDC and Infectious disease database of hospitals in six provinces and cities in China from 1999 to 2015. All patients with a diagnosis of ‘MDR spinal TB’ were diagnosed by culture or gene chip combined with clinical symptoms and imaging characteristics. The available hospital records were reviewed. Documented admission information included the following: age, sex, region, educational status, symptom, erythrocyte sedimentation rate (ESR), drug resistance, therapeutic regimen, the mean time from symptom occurrence to diagnosis, length of stay, comorbidities and adverse drug reaction. For patients who underwent spinal surgery, the following complications were recorded: delayed paralysis, sinus, incision disunion, instrumentation failure and recurrence.

### Therapeutic regimen

All patients underwent four drug anti-tuberculous chemotherapy HREZ (rifampicin, 15 mg/kg, maximum, 600 mg/day; and isoniazid, 6 mg/kg, maximum 300 mg/day and ethambutol, 25 mg/kg, maximum 750 mg/day and pyrazinamide, 25 mg/kg, maximum 750 mg/day) for at least 18 months. In order to improve the compliance and convenience of patients outside hospital, all patients and their families received education and training, made clear the importance of early, regular, appropriate, whole-course and combined medication, supervise patients medication. Conservative treatment patients are performed lying in bed, to avoid strenuous exercise. The surgical protocols included the anterior approach, the posterior approach and the combined anterior posterior approach. One-stage anterior surgical indication includes the damaged portion of the vertebra being located in the anterior and middle columns of the spine; the abscess or sequestrum invading the anterior spinal canal and causing compression; unapparent kyphosis; laminectomy having been performed and posterior bone graft fusion being prevented and when the number of damaged vertebrae is less than 3. Posterior-only approach surgical indications include the lesion being confined to less than three adjacent segments, or, if multiple segments are involved, only one or two vertebra needing to be addressed surgically, there is normal bone to be used as a tunnel for internal fixation, patients having previously undergone anterior surgery and the structure not being clear; complete debridement can be achieved through the posterior-only approach, with long bone segments not being needed to restore the anterior and middle column heights after debridement. Anterior combined posterior approach indications include severe kyphosis can’t be corrected by anterior or posterior approach, severe spinal instability after the debridement and extensive paravertebral abscess or sequestrum, the number of severely damaged vertebral bodies is more than 3. The surgical approach should be selected according to the properties of the infection type, pathological changes, lesion scope and general physical condition. The surgical procedures were performed according to standard surgical approaches and then exposure focus, completely debridement, bone graft fusion and instrumentation, repetitious irrigating. Finally, treatment with 1.0 g of streptomycin and 0.3 g of isoniazid was locally administered; drainage and incision sutures were performed post-operatively. The patient can't perform the anterior approach surgery or has previously failed anterior approach surgery. Simultaneously, it should not be used for patients who are in poor systemic conditions and can't tolerate surgery or suffered serious lesions of other organs. Simple debridement or computed tomography (CT)-guided puncture drainage, local chemotherapy indications include no obvious instability and neurological dysfunction, abscess exceed two vertebrae, fluid abscess, sinus or dead bone, simple spinal adnexal TB. The change of the patient's vital signs should be observed 3 days after the operation, and antibiotics were used to prevent infection 3 days after operation. One gram rifamycin sodium or 0.6 g isoniazid was added to 500 ml saline for 24 h with continuous infusion. The drainage time was 3 weeks to 3 months.

### Evaluation index

Clinical symptoms, signs and nerve function improvement were observed. The erythrocyte sedimentation rate (ESR), liver and kidney function were monitored. Radiology evaluated correction of kyphosis and bone fusion. The visual analogue scale (VAS) was used to assess pain improvement and neurological status was assessed according to the American Spinal Injury Association (ASIA) impairment scale.

Microbiological diagnosis includes at least one of the following: *M. tuberculosis* in blood, bone, deep soft tissues and/or abscesses; positive microscopy for acid-fast bacilli from bone, deep soft tissue, psoas or paravertebral abscess (Ziehl–Neelsen staining) and positive rapid culture system results, such as BACT/MGIT 960 system, the absolute concentration method on L–J medium and DNA microarray for *M. tuberculosis* complex. Nucleic acid amplification tests adopted the Capital-Bio™ DNA Microarray assay. Drug susceptibility tests adopted the agar proportion method, Capital-Bio™ DNA Microarray assay Genotype MTBDR plus assay.

### Statistical analysis

Demographic and clinical characteristics were collected and included gender, age, place of residence, degree of education, neurological deficit and ESR. All variables were tested for association with MDR spinal TB using univariate logistic analysis. Associations between MDR spinal TB and predictor variables were expressed using odd ratios (ORs) with a 95% confidence interval (95% CI). The *χ*^2^ test or Fisher's exact test was used to examine surgery frequency between the culture methods group and molecular diagnosis group. The paired *t* test was used to compare the preoperative and postoperative degree of ESR and VAS levels. The independent sample *t* test was used to assess the between-group differences with respect to various laboratory and physical parameters. The Wilcoxon signed rank test was used to compare preoperative and postoperative ASIA classification. *P*-Values of <0.05 were considered to indicate statistical significance. All statistical analyses were performed using SPSS version 20.0 (SPSS Inc, Chicago, USA).

## Results

From 1999 to 2016, the number of spinal TB patients per year stably decreased, but the number of MDR spinal TB cases increased. In this study, there were 272 MDR spinal TB patients, comprised of 85 women and 187 men, including 8 children and 264 adults. The average age was 31.16 ± 15.43 years, with the male patients having an average age of 29.64 ± 13.61 years, and the female patients being aged 32.38 ± 11.73 years. The largest number of patients fell in the 15–30 age group (34.19%). Urban patients accounted for 58 cases (21.32%), and rural patients accounted for 214 cases (78.68%); there were 56 cases (cities) and 150 cases (rural) with a middle school education and above. The mean time from symptom occurrence to diagnosis was 15.73 months in rural patients and 11.41 months in urban patients. Neurological deficit was seen in 83 patients (30.51%). ESR is a routine investigation which ranged from 1–127 mm/h in our study. The mean ESR was 50.94 mm/h. The percentage of patients with ESR less than 20 mm/h was 10.66%. The percentage of patients with ESR more than 100 mm/h was 33.82%. ESR returned to normal by the last visit in all patients (Table 1).

**Table 1.** Demographic and clinical characteristics and logistic regression model analysis of variables associated with MDR spinal TB patients in this study

Characteristics	*N* (%)			
	Spinal TB* *N* = 2865	MDR spinal TB *N* = 272	OR (95% CI)	*P* value
Sex				
Male	1466 (51.17)	187 (68.75)	1.00	−
Female	1399 (48.83)	85 (31.25)	0.48 (0.37–0.62)	0.00
Age				
<14	145 (5.06)	8 (2.94)	0.64 (0.30–1.36)	0.24
15–30	1089 (38.01)	93 (34.19)	0.99 (0.72–1.37)	0.95
31–40	799 (27.89)	69 (25.37)	1.00	−
41–50	513 (17.91)	47 (17.28)	1.06 (0.72–1.56)	0.77
51–60	205 (7.15)	34 (12.5)	1.92 (1.24–2.98)	0.00
>60	114 (3.98)	21 (7.72)	2.13 (1.26–3.61)	0.00
Region				
Urban	936 (32.67)	58 (21.32)	1.00	−
Rural	1929 (67.33)	214 (78.68)	1.79 (1.33–2.42)	0.00
Education				
Primary	973 (33.96)	66 (24.26)	0.74(0.55–1.00)	0.05
Middle	1596 (55.71)	146 (53.68)	1.00	−
University	296 (10.33)	60 (22.06)	2.22 (1.60–3.07)	0.00
Neurological deficit				
Yes	1009 (35.22)	83 (30.51)	1.00	−
No	1856 (64.78)	189 (69.49)	1.24 (0.95–1.62)	0.12
ESR (mm/h)				
Normal (<20)	407 (14.21)	29 (10.66)	1.06 (0.70–1.60)	0.78
Moderate–elevated (20–100)	2250 (78.53)	151 (55.51)	1.00	−
High-elevated (>100)	208 (7.26)	92 (33.82)	6.59 (4.90–8.86)	0.00

MDR: multi-drug resistant; TB: tuberculosis; ESR: erythrocyte sedimentation rate.

*Spinal TB without MDR.

On univariate analysis, compared with spinal TB patients without MDR, MDR spinal TB showed a lower proportion of women (OR 0.48, 95% CI 0.37–0.62, *P* < 0.01), a large proportion of rural population (OR 1.79, 95% CI 1.33–2.42, *P* < 0.01) and higher education attainment (OR 2.22, 95% CI 1.60–3.07, *P* < 0.01). Using the group of patients aged 31–40 years as a reference, the percentage of MDR cases within the ⩾51 years age group significantly exceeded that of TB cases without MDR (OR 1.92, 95% CI 1.24–2.98, *P* < 0.01), while no significant differences in MDR *vs.* TB case percent rates were noted for groups of patients aged <14 years and 15–30 years (*P* > 0.05). In addition, patients with MDR spinal TB were significantly more likely to exhibit high-elevated ESR than those with pulmonary TB diseases (OR 6.59, 95% CI 4.90–8.86, *P* < 0.01). Meanwhile, no other differences were found based on neurological deficit between two groups (*P* > 0.05).

Back pain is the most common symptom, followed by low fever and night sweats. Upon physical examination, there were 194 cases with spinal activity limitation, 257 cases with local percussion pain, 72 cases with kyphosis and 53 cases with skin ulceration and sinus formation. There were 83 patients with neurological symptoms, including radicular pain in 49, numbness in 43, weakness in 36 and trouble walking in 28 (Table 2).

**Table 2.** Clinical features at presentation (*n* = 272)

Features	Number of patients (%)
Back pain	268 (98.53)
Low fever or/and night sweats	260 (95.59)
Local percussion pain	257 (94.49)
Spinal activity limitation	194 (71.32)
Kyphosis	72 (26.47)
Skin ulceration or/and sinus formation	53 (19.49)
Neurological symptoms	83 (30.51)
Radicular pain	49 (18.01)
Numbness	43 (15.81)
Weakness	36 (13.24)
Trouble walking	28 (10.29)

Through traditional bacterial culture, drug sensitivity test and gene chip test, we observed the proportion of drug-resistant TB increased year by year. Among all TB patients resistant to first-line drugs, isoniazid resistance was the highest, for 187 cases, followed by rifampicin, for 156 cases and then streptomycin, for 114 cases. The most common second-line drug resistance was levofloxacin (79 cases), followed by rifapentine (62 cases), with the least common being Amikacin (23 cases) and kanamycin (15 cases) (Table 3). There were 272 consecutive culture-confirmed MDR spinal TB patients whose laboratory test information was intact. A total of 188 (69%) were diagnosed by standard culture and drug susceptibility test methods (Culture methods), and 84 (31%) were diagnosed following implementation of detection using molecular diagnosis. The patients diagnosed by molecular biology began the rational use of second-line drugs earlier than patients diagnosed by culture methods (6 days *vs.* 74 days). Among patients who were admitted to a general TB ward, those diagnosed by molecular methods spent less time in the ward designated for patients with drug-susceptible infections and less total days of hospitalisation than those in diagnosed by culture methods (5 days *vs.* 35 days and 20 days *vs.* 34 days, respectively). The use of molecular diagnosis resulted in noteworthy clinical advances, including earlier initiation of MDR spinal TB treatment, improved infection control and better clinical outcome. All patients diagnosed using molecular technology were eventually hospitalised for MDR spinal TB therapy and placed in drug-resistance sickrooms. In contrast, patients diagnosed by standard culture and drug susceptibility test methods were placed in sickrooms designated for patients with drug-susceptible infections. Of the 272 inpatients with MDR spinal TB, 138 patients started extramural hospital treatment using first-line drugs before their drug susceptibility test results were known. Of these 138 patients, the mean time until hospitalisation and starting second-line drug pharmacotherapy was 75 days in diagnosed by molecular methods compared with 128 days in patients diagnosed by culture methods (Table 4).

**Table 3.** Characteristics of MDR spinal TB

	Patients (272)		Patients (3137)
	*N*	%	%
MDR-STB	272	100	8.67
First-line drugs			
Isoniazid	187	68.75	5.96
Rifampicin	156	57.35	4.97
Streptomycin	114	41.91	3.63
Second-line drug			
Levofloxacin	79	29.04	2.52
Rifapentine	62	22.79	1.98
Amikacin	23	8.46	0.73
Kanamycin	15	5.51	0.48

MDR: multi-drug resistant; TB: tuberculosis.

The first column represents the patients with MDR spinal TB (272); the second column represents all patients with spinal TB (3137). The data is the ratio of the number of patients with each drug resistance to the total number of patients. For example: 272/3137 = 8.67%; 187/272 = 68.75%; 187/3137 = 5.96%. Because each patient is resistant to two or more drugs, the sum of the number of patients who were resistant to each drug exceeds the total number (272).

**Table 4.** Therapeutic outcome of MDR spinal TB patients diagnosed using conventional culture and molecular diagnosis technology (*n* = 272)

Characteristic	Overall	Culture methods	Molecular diagnosis
	Value		
Treatment with first-line drug regimen *N* (%)	272 (100)	188 (69)	84 (31)
Mean days on first-line drug*	81 [14–434 d]	154 [73–434 d]	24 [14–38 d]
Mean days until starting treatment using second-line drug*	44 [2–102 d]	74[54–102 d]	6 [2–7 d]
Mean days in drug-susceptible sickroom*	19 [3–59 d]	35[17–59 d]	5 [3–7 d]
Mean days of drug treatment prior to hospitalisation*	49 [13–108 d]	84 [16–367 d]	18 [13–31 d]
Mean days until hospitalisation and starting second-line drug pharmacotherapy* (*N* = 138) #	111 [31–365 d]	128[31–365 d]	75 [31–332 d]
Total days of hospitalisation*	25 [11–59 d]	34 [17–59 d]	20 [11–34 d]
Required surgery* *N* (%)	159 (58)	114 (61)	45 (54)
Recovered without need for surgery *N*	8	0	8

MDR: multi-drug resistant; TB: tuberculosis.

**#**: *n* = 138: MDR spinal TB patients started extramural hospital treatment using first-line drugs before their drug susceptibility test results were known.

*P < 0.05. Comparison between the culture methods group and molecular diagnosis group.

In total, 106 patients only underwent conservative treatment, and 159 patients had combined surgical treatment with drug chemotherapy. In addition, seven patients underwent local CT-guided puncture and local chemotherapy. All three therapeutic regimens could yield satisfactory outcomes (Table 5). In terms of operation results, the correction rate of kyphosis was up to 80%, and the bone fusion rate was up to 80% to 90% at 1 year follow-up. In total, 39 patients developed postoperative complications, mainly at one week to one year after surgery. Here, 17 patients had postoperative sinus formation, which was cured by surgery. There were five patients with delayed paralysis who later suffered revision surgery. There were 11 patients with incision disunion, who were healed by local dressing change or skin grafts, and two patients suffered instrumentation fracture, which was resolved after revision surgery. There were 14 cases of recurrence, who underwent reoperation. There were nine patients with local abscesses, treated with incision, drainage and local or whole body anti-TB drugs, and five patients showed new abscesses at the non-surgical section, who were treated with surgery. In addition, 21 had drug-related complications, including gastrointestinal symptoms (13), followed by liver injury (6), then peripheral nerve damage (1) and hearing impairment (1) (Table 6).

**Table 5.** Treatment and follow-up outcomes of 272 MDR spinal TB patients in this study

Treatment	ESR (mm/h)	VAS	ASIA (*N*)				
			A	B	C	D	E
Only chemotherapy (*N* = 106)							
Prior treatment	51.41 ± 23.42	5.46 ± 1.76	0	0	2	8	96
Final follow-up	5.93 ± 3.22*	1.04 ± 0.98*	0	0	0	5	101*
Chemotherapy + anterior approach (*N* = 39)							
Pre-operation	48.13 ± 27.45	5.71 ± 1.58	2	1	2	7	27
3 months postoperative	6.93 ± 4.42*	2.44 ± 0.78*	0	0	2	1	36*
Final follow-up	5.29 ± 3.03^Δ^	0.47 ± 0.63^Δ^	0	0	1	1	37
Chemotherapy + posterior approach (*N* = 68)							
Pre-operation	50.53 ± 25.98	5.89 ± 1.68	3	0	7	25	33
3 months postoperative	7.09 ± 4.23*	2.66 ± 0.95*	0	1	3	10	54*
Final follow-up	5.88 ± 2.93^Δ^	0.61 ± 0.63^Δ^	0	0	2	2	64^Δ^
Chemotherapy + combined approach (*N* = 52)							
Pre-operation	46.79 ± 22.26	5.88 ± 1.91	5	0	5	16	26
3 months postoperative	6.62 ± 4.61*	2.67 ± 0.91*	0	0	5	2	45*
Final follow-up	5.71 ± 1.58	0.48 ± 0.60^Δ^	0	0	4	1	47
Chemotherapy + CT guided puncture (*N* = 7)							
Pre-operation	52.46 ± 25.34	5.88 ± 1.91	0	0	0	3	4
3 months postoperative	7.14 ± 3.81*	1.67 ± 0.79*	0	0	0	1	6*
Final follow-up	6.22 ± 4.11	1.27 ± 0.65	0	0	0	0	7

ESR: erythrocyte sedimentation rate, VAS: Visual Analogue Scale, ASIA: American Spinal Injury Association grading.

*P < 0.05 *vs.* pre-operation or prior treatment.

Δ*P* < 0.05 *vs.* 3 months postoperative.

**Table 6.** Imaging findings and clinical treatment of MDR spinal TB

Characteristic	*N* (%)
Site	272 (100)
Cervical	16 (5.88)
Cervicothoracic	5 (1.84)
Thoracic	97 (35.66)
Thoracolumbar	43 (15.81)
Lumbar	85 (31.25)
Lumbosacral	26 (9.56)
Number of vertebrae involved	272 (100)
1–2	199 (73.16)
3–4	54 (19.85)
⩾5	19 (6.99)
Treatment	272 (100)
Only Chemotherapy	106 (38.97)
Chemotherapy + Local CT guided puncture	7 (2.57)
Chemotherapy + Operation	159 (58.46)
Drug-related complications	21 (7.72)
Gastrointestinal symptoms	13 (4.78)
Liver injury	6 (2.21)
Peripheral nerve damage	1 (0.37)
Hearing impairment	1 (0.37)
Postoperative complications	39 (24.53)
Sinus formation	17 (10.69)
Delayed paralysis	5 (3.14)
Incision disunion	11 (6.92)
Instrumentation fracture	2 (1.26)
Recurrence	14 (8.81)
Local abscesses	9 (5.66)
New abscesses at the non-surgical section	5 (3.14)

MDR: multi-drug resistant; TB: tuberculosis.

CT: computed tomography.

## Discussion

TB is an enormous threat to human health. Pott first described spinal TB in 1782 [8]. In developing countries, spinal TB occurs in older children and young adults, especially in poverty-associated regions, while in developed countries it has become a disease of older patients [9,10]. Moghtaderi *et al*. reviewed 43 patients with S-TB; the mean age of patients was 45.4 ± 21 years (range, 10–75 years) and 70% of the patients were between 35 and 55 years [11]. In an epidemiological survey of spinal TB from 2002 to 2011 in the United States, Ramos pointed out the mean age of patients was 51 years (IQR, 35–65 years); those aged over 65 years accounted for 24.5% [12]. In another study, 55% of patients were older than 70 years [13]. In this research, MDR spinal TB can occur at any age, with a minimum age of 2 years, a maximum age of 89 years and an average age of 33.91 years. The largest group of patients was 15 to 30 years old. We found that MDR spinal TB patients aged over 51 years have a higher proportion than spinal TB without MDR. This increase in older patients may be associated with the poor immunological function, and the increased number of comorbidities in this age group. The current study found a higher incidence of MDR spinal TB in rural populations than in urban populations and spinal TB patients with high educational attainment might be more vulnerable to developing MDR spinal TB. Overall, 78.68% of patients in this research lived in rural areas, which are associated with poor economic level and poor medical and health conditions. Patients with middle school education and above accounted for 70.09% in rural and 96.55% in urban areas. In a retrospective study, Wang *et al*. found that the proportion of the rural population was 59.5%, and that patients with middle school education and above accounted for 60.4% in rural and 68.7% in urban areas[14]. Developments in transportation and increases in population mobility may have contributed to this change. In high-incidence areas, spinal TB is frequently associated with pulmonary TB, with a proportion of 14.3–32% [12–14]. Therefore, when spinal TB is suspected, sputum samples and chest radiography should be performed wherever possible in order to avoid misdiagnosis of the associated pulmonary TB, delay treatment or even risk infection transmission. In this research, there were 19 cases of accompanied pulmonary TB, which was lower than previous studies.

The current study also found that women were associated with a lower risk of MDR spinal TB. The male provides the main labour force, which is important in that long-term physical labour increases the load on and activity of the spine, resulting in chronic spinal damage, thus increasing susceptibility to MDR spinal TB. Assessment of ESR among patients with spinal TB is common. In this study, patients with MDR spinal TB were significantly more likely to exhibit high-elevated ESR than those with spinal TB diseases without MDR. Although it is used only to evaluate disease activity, based on these results and the authors' experience, high-elevated ESR occurred in spinal TB patients is highly suggestive of MDR spinal TB.

Spinal TB is characteristically chronic and slowly progressive. Back pain is the most common symptom, followed by night sweats and low-grade fever. Night sweats and mild fevers do not happen continually, but these symptoms are persistent according to the patient's medical history. This characteristic may be related to the latent clinical course and nonspecific clinical manifestations. According to the clinical manifestation and physical examination, we found that limited spinal motion, percussion pain and kyphosis were the main clinical signs, and neurological deficits occurred less frequently. McLain *et al*. reported that neurological deficits are common, with long-term thoracic and cervical involvement, and if left untreated, neurological deficits may progress to complete and incomplete paraplegia [15]. In our study, there were 523 patients with neurological deficits, accounting for 37.30% of cases. At the last follow-up, neurological function improved to different degrees.

ESR is a routine test target, with an average of 84.5 and 43.5 mm/h reported in the literature [16 17]. Our results showed ESR ranges from 1 to 127 mm/h, with a mean value of 50.94 mm/h. ESR has high sensitivity, but low specificity, and is primarily used to evaluate disease activity during follow-up.

Anti-TB chemotherapy is the basis for the treatment of spinal TB. Without medication, the surgical risk and complications increase significantly. Simultaneously, the mortality rate and the risk of infection transmission also increase [18]. However, the duration of drug treatment is still controversial. Some scholars believe that drug chemotherapy is required for 6 months, combined with surgery, which is the same as undergoing simple chemotherapy for 9 months. The American Thoracic Society recommends 6–9 months of chemotherapy for spinal TB patients, and some studies have shown that this regimen has a favourable clinical outcome [19, 20]. Wang [21] treated patients with ultra-short range chemotherapy combined with surgery and also achieved good results. In this research, all MDR spinal TB patients were given chemotherapy for 18–26 months. Optimal management of MDR spinal TB relies on the early detection of the disease in such patients [22]. In this research, we observed improved therapeutic effects in patients in whom the gene chip was used for diagnosis. For example, local symptoms can be eased significantly or even disappear with the use of medication, thus avoiding surgery. Almost all of these cases occurred in molecular diagnosis group, which was superior to the culture methods group in clinical outcome. In addition, more patients in the molecular diagnosis group accessed surgical treatment at an earlier time.

Surgical indications include spinal cord compression and/or injury, nerve root compression, severe kyphosis, spinal instability, progressive kyphosis and important organs compression. In this study, the larger psoas muscle and/or paravertebral abscess was not an indication for surgery; some patients (383 cases) were cured by drug therapy or CT-guided puncture combined with local drug chemotherapy, avoiding open surgery. For patients who met the absolute surgical indications, early surgical treatment is recommended on the basis of effective chemotherapy, not only to promote the recovery of neurological function and control kyphosis over time, but because they can also get to the pathological tissue earlier, acquire drug sensitivity data earlier and develop subsequent individual chemotherapy protocols [23]. For patients with relative surgical indications (sequestrum, sinus and localised abscess), we should choose an individual treatment according to the patient's specific condition [24, 25].

In recent years, increasing drug-resistant TB, especially MDR spinal TB, has become a big problem for surgeons. Because drug-resistant TB has the characteristics of a long onset time, wide range of lesions and easy recurrence, it is very important to adopt the complete debridement and stable internal fixation. The operative indications should be strictly controlled for the DR-TB, and individualised surgical procedures should be developed according to the location, nature and extent of the lesion [26,27]. In the study, 137 drug resistance patients underwent surgical treatment combined with individualised chemotherapy and achieved clinical cure. Some researchers have paid close attention to nosocomial transmission and to the cross-infection of patients with drug-susceptible TB by patients with MDR spinal TB [13,14]. Using molecular probe technology to detect TB, we are able to rapidly diagnose MDR spinal TB and begin the use of second-line anti-TB drugs as soon as possible. At the same time, we can control TB nidus, achieve earlier surgical treatment for spinal TB, shorten patients' hospitalisation times and improve the effects of drug treatments.

Since China formulated the national plan for the prevention and cure of TB in 1991, there has been a great deal of progress in TB control. At present, the standard of dots mainly uses HREZ regimens in clinical treatment. However, all drugs can cause varying degrees and frequencies of adverse reactions; thus the patient's adherence to treatment will be reduced, which will directly affect TB prevention and control. With the duration of medication increased, the incidence of adverse reactions to anti-TB drugs will increase significantly. In our study, the most common adverse drug reactions were gastrointestinal symptoms, followed by liver injury and this was similar to the results of other articles [4,17].

The present study has several limitations. First, this study is a multicentre retrospective study based on medical records, different hospitals have different specialties and abilities, which may cause a certain degree of bias. Second, in the analysis of the risk factors for drug-resistant spinal TB, we used the non-drug-resistant spinal TB population as the control group, not the healthy population. Therefore, further study is needed to collect the community resident's information and to analysis risk factors for MDR spinal TB. Lastly, molecular diagnostic technology has only been widely applied in recent years, and it is mainly applied in hospitals in economically developed areas. This study has a large time span and a wide geographical range, this may have resulted in a bias in outcomes of molecular diagnostic application.

## Conclusions

MDR spinal TB is still a severe problem threatening public health. Back pain is the most common clinical symptom, and the thoracic spine is the vertebral body most often involved. The most common first-line and second-line resistant drug was isoniazid and levofloxacin, respectively. Rural district, advanced in years, high education degree in spinal TB patients should be focused on preventing the occurrence of MDR spinal TB. The use of molecular diagnosis resulted in noteworthy clinical advances and it may be employed for better diagnosis, management and treatment of MDR spinal TB. This study contributes valuable information about the epidemiological characteristics and influence factors of MDR spinal TB of the period in six provinces and cities of China, thus increase attention and knowledge of this disease in the region and the country.
